# The Use of Urotropin in Acute Inflammaions of the Middle Ear

**Published:** 1911-11

**Authors:** F. Phinizy Calhoun

**Affiliations:** Atlanta, Ga.


### THE USE OF UROTROPIN IN ACUTE INFLAMMATIONS OF THE MIDDLE EAR.

F. Phinizy Calhoun, A. B., M. D., Atlanta, Ga.

It is with an apology that I present to you an article on the uses of Urotropin, yet with an experience extending over one year, in which Urotropin was given for acute inflammations of the middle ear, this paper might be of interest to a few.

It is to Crowe, a native of this city, that we owe a debt of gratitude for his untiring and painstaking research work on animals and man at the Johns Hopkins Medical School, and presenting his experiments to us in such a clear and concise manner.

The drug was first prepared in 1860 by Butlerow, and it is made by the action of ammonia on formaldehyde according to the following equation:

$$4NH_3+6HCHO=(CH_2)_6N_4+6H_2O.$$

Urotropin readily yields ammonia and formaldehyde on boiling with dilute acid, in fact, in neutral aqueous solutions it likewise decomposes when heated, and for that reason the equation is considered a reversible one. The temperature of the body and the reaction of the body fluids might effect the liberation of its component parts. It was first introduced into therapeuttics in 1894 by Nicolier, under the name of Urotropin, and was made official in the U. S. Pharmacopoeia in 1900 under the name of Hexamethylenamin.

Richardson and Churchman in this country were among the first to recommend its use in typhoid fever and as an urinary antiseptic.

The detection of formaldehyde in the secretion is made by many tests, that of Hehner is the one recommended by Crowe, and its modification by him is as follows:

Four to six drops of milk are added to a few c. c. of the material to be tested and this mixture is stratified with an equal volume of the reagent, which is composed of 100 c. c. of 90% sulphuric acid and one drop of 3% ferric chloride soluton. The sulphuric acid decomposes the Hexamethylenamin into ammonia and a deep amethyst color develops at the junction of the layers.

In a conversation with Dr. Crowe about Christmas, 1909, and later in a communication, he suggested that Urotropin might be of value in middle ear inflammations, although he had made no investigation of its use in middle ear discharges.

Acting upon his advice I commenced to use it in acute catarrhal and purulent inflammations of the middle ear. In my early cases, smears, often cultures, were first made of the aural discharges to determine the nature of the infection, then Urotropin was given somewhat after the manner prescribed by Crowe, viz.: 100 grains dissolved in 1000 c. c. of water, and to the patient this was given in 24 hours to toleration, carefully watching for haematuria.

Dr. J. E. Paullin, of the State Board of Health, gave me every assistance in examing the aural secretions, using Helmer's test to detect formaldehyde. These examinations were also carried out at the laboratory of Grady Hospital.

A few of the cases which led me to believe that Urotropin was of some value in acute middle ear infections are briefly mentioned.

Case 1. Miss B. Pain in right ear day before consultation. After a restless night with earache she woke up next morning feeling much relieved, with watery discharge from the ear. The examination showed the canal contained bloody serum and from a very small perforation in the posterior interior quadrant of the drum, which was only slightly injected, came a fluid of the same character. Smear and stain showed diplococci. I prescribed a cathartic, hot saline irrigations and Urotropin tablets, 7 grains every two hours. Patient came to my office late next day feeling better except for a slight deafness. The canal was then dry and only a slight hyperaemia of the drum. Same treatment continued, except Urotropin reduced; the following day the tympanum was inflated and the patient dismissed.

Case 2. Baby S., age two years, seen January 7th, 1910. There had been a muco-purulent discharge from the right ear for two weeks. For several days previous there had been more or less septic temperature and sweats. The examination showed a small perforation in the anterior inferior quadrant and the canal contained a ropy discharge. The pneumococcus was the offending organism. Prescribd saline irrigations. Urotropin solution equivalent to 3 grains every three hours. January 9th, much better, no temperature, discharge less. January 14th, no discharge and dismissed.

Case 3. Katherine H., age four and one-half years, seen first June 27th, 1910; purulent discharge from the right ear for three weeks following measles. The organism found was a diplococcus. The canal contained much pus and the drum looked rather angry. Urotropin, 3 grains, was given and saline irrigations. No discharge in four days.

Case 4. John S., age thirteen. Acute purulent otitis media of one month's duration. Three days before I saw him he developed pain behind ear and much headache. The parents had consulted an aurist who advised an immediate operation for acute mastoiditis. I was consulted September 14th, 1910, and found the patient with a temperature of 101, very slender tip, profuse aural dscharge, good opening in drum. The infection was staphylococcis aureus, detected from culture made by Dr. Paullin. Sent to hospital for observation, and prescribed calomel, rest in bed, saline irrigations and Urotropin solution 7 grains q. 3 h. September 15th, no tenderness, temperature not above 99 in twenty-four hours. Urotropin increased to q. 2 h. September 22nd discharge ceased and dismissed in a few days.

These cases represent some of the cures aided, so I considered by Urotropin. There were others in which no results were obtained. In a few cases I prescribed Urotropin for a chronic purulent otitis media, without results, except that the odor was somewhat reduced.

To gain assistance in the investigation of the use of Urotropin in the treatment of acute otitis media, I wrote to an acquaintance, Dr. Walters, House Surgeon at one of the large New York ear hospitals, suggesting that he use it in appropriate cases. He had at his command a greater number of cases and I considered his opinion to be of value, yet his conclusions were entirely opposite to mine in that he noticed no appreciable effect.

Soon after I commenced my investigation there appeared in the Journal of the A. M. A. (Vol. Liv. No. 11), an article by Bartow, the first so far as I am able to investigate, on the elimination of Urotropin by the mucosa of the middle ear, describing a case of acute otitis media of pneumococci infection, cured in two days following average doses of Urotropin.

At the present time Urotropin is frequently used by aurists in inflammations of the middle ear, but with what results, I do not know. The conclusion that I have made in using Urotropin in perhaps fifty cases of acute or sub-acute otitis media is that the duration of the discharge is apparently shortened, and the opening of the drum heals with more rapidity; I am free to admit, however, that I have no positive proof that Urotropin causes this.

Again, the nature of the infection undoubtedly places considerable importance in the resistance to Urotropin. Cases of otitis media of staphylococcic and pneumococcic origin, respond more quickly than when due to a more violent organism, such as the streptococcus.

I believe that Crowe's experiments in meningitis have been entirely different.

Finally, Urotropin is a harmless drug. Its only unpleasant symptom of an overdose is haematuria, which can easily be controlled.

